# A comparative transcriptomic analysis of Glucagon-like peptide-1 receptor- and Glucose-dependent insulinotropic polypeptide receptor-expressing cells in the hypothalamus

**DOI:** 10.1016/j.appet.2022.106022

**Published:** 2022-04-14

**Authors:** Christopher Smith, Ryan Patterson-Cross, Orla Woodward, Jo Lewis, Davide Chiarugi, Florian Merkle, Fiona Gribble, Frank Reimann, Alice Adriaenssens

**Affiliations:** 1 Wellcome Trust-MRC Institute of Metabolic Science, Addenbrooke’s Hospital, Cambridge, UK; 2 Max Planck Institute for Human Cognitive and Brain Sciences, Leipzig, Germany

**Keywords:** Gut-brain axis, hypothalamus, transcriptomics, feeding, appetite, Glucagon-like peptide-1, Glucose-dependent insulinotropic polypeptide

## Abstract

**Objective:**

The hypothalamus is a key region of the brain implicated in homeostatic regulation, and is an integral centre for the control of feeding behaviour. Glucagon-like peptide-1 (GLP-1) and glucose-dependent insulinotropic polypeptide (GIP) are incretin hormones with potent glucoregulatory function through engagement of their respective cognate receptors, GLP-1R and GIPR. Recent evidence indicates that there is a synergistic effect of combining GIP- and GLP-1-based pharmacology on appetite and body weight. The mechanisms underlying the enhanced weight loss exhibited by GIPR/GLP-1R co-agonism are unknown. *Gipr* and *Glp1r* are expressed in the hypothalamus in both rodents and human. To better understand incretin receptor-expressing cell populations, we compared the cell types and expression profiles of *Gipr*- and *Glp1r*- expressing hypothalamic cells using single-cell RNA sequencing.

**Methods:**

Using *Glp1r*-Cre or *Gipr*-Cre transgenic mouse lines, fluorescent reporters were introduced into either *Glp1r*- or *Gipr*-expressing cells, respectively, upon crossing with a *ROSA26*-EYFP reporter strain. From the hypothalami of these mice, fluorescent *Glp1r*^EYFP+^ or *Gipr*^EYFP+^ cells were FACS purified and sequenced using single-cell RNA sequencing. Transcriptomic analysis provided a survey of both non-neuronal and neuronal cells, and comparisons between *Glp1r*^EYFP+^ and *Gipr*^EYFP+^ populations were made.

**Results:**

A total of 14,091 *Glp1r*^EYFP+^ and *Gipr*^EYFP+^ cells were isolated, sequenced and taken forward for bioinformatic analysis. Both *Glp1r*^EYFP+^ and *Gipr*^EYFP+^ hypothalamic populations were transcriptomically highly heterogeneous, representing vascular cell types, oligodendrocytes, astrocytes, microglia, and neurons. The majority of *Gipr*^EYFP+^ cells were non-neuronal, whereas the *Glp1r*^EYFP+^ population was evenly split between neuronal and non-neuronal cell types. Both *Glp1r*^EYFP+^ and *Gipr*^EYFP+^ oligodendrocytes express markers for mature, myelin-forming oligodendrocytes. While mural cells are represented in both *Glp1r*^EYFP+^ and *Gipr*^EYFP+^ populations, *Glp1r*^EYFP+^ mural cells are largely smooth muscle cells, while the majority of *Gipr*^EYFP+^ mural cells are pericytes. The co-expression of regional markers indicate that clusters of *Glp1r*^EYFP+^ and *Gipr*^EYFP+^ neurons have been isolated from the arcuate, ventromedial, lateral, tuberal, suprachiasmatic, and premammillary nuclei of the hypothalamus.

**Conclusions:**

We have provided a detailed comparison of *Glp1r* and *Gipr* cells of the hypothalamus with single-cell resolution. This resource will provide mechanistic insight into how engaging *Gipr* and *Glp1r* cells of the hypothalamus may result in changes in feeding behaviour and energy balance.

## 1 Introduction

The hypothalamus is a key region of the brain implicated in homeostatic regulation, and is integral to the tight control of an organism’s energy balance. Studies in preclinical models have identified key circuits within the hypothalamus that integrate nutritive and metabolic cues from the periphery with neural networks that regulate appetite, feeding behaviour, and energy expenditure (Yeo & Heisler, 2012). This circuitry lies within discrete nuclei of the hypothalamus that are critical for maintaining energy homeostasis (Gao & Horvath, 2007). In addition to neuronal cells within these nuclei, it is becoming increasingly apparent that non-neuronal cells share in the task of integrating information regarding an organism’s metabolic state (Butiaeva et al., 2021; Djogo et al., 2016; Kohnke et al., 2021; Reiner et al., 2016). With increasing genetic evidence indicating that obesity is driven by aberrations in signalling pathways of the central nervous system (CNS), cell surface receptors and protein effectors involved in signalling cascades acting through different cell types located within these hypothalamic nuclei may represent promising targets for the development of therapeutics (Locke et al., 2015).

Glucagon-like peptide-1 (GLP-1) and glucose-dependent insulinotropic polypeptide (GIP) are incretin hormones with potent stimulatory effects on postprandial insulin release (Drucker, 2018). GLP-1 receptor (GLP-1R) agonism is an established therapeutic strategy for treating and managing type 2 diabetes (T2D). In addition to bringing glucoregulatory benefit, the GLP-1 receptor (GLP-1R) agonist (GLP1-RA) drug class has recently been licensed for the treatment of obesity, owing to the anorexigenic properties of GLP-1R engagement in the CNS (Gabery et al., 2020; Secher et al., 2014; Wilding et al., 2021). Central mechanisms underlying the effect of GLP-1R agonism on body weight and feeding are thought to involve recruitment of pathways triggering malaise and emesis in the dorsal vagal complex and parabrachial nucleus, reducing the reward value of food through engaging the mesolimbic reward system, as well as further suppressing appetite through activating hypothalamic nuclei (Alhadeff et al., 2014; Alhadeff et al., 2012; Borner et al., 2021; Richard et al., 2014; Secher et al., 2014).

The next generation of therapeutics for the treatment of metabolic disease includes multifunctional GLP-1RA-based drugs designed with the aim to simultaneously target multiple signalling pathways to improve the therapeutic index. The GIP receptor (GIPR) signalling axis has proven to be an effective co-target, with both GIPR agonists and antagonists demonstrating enhanced weight loss or prevention of weight gain when combined with GLP-1R agonism in preclinical models (Coskun et al., 2018; Lu et al., 2021). The advancement of GIPR/GLP-1R dual agonists to late-stage clinical trials has further highlighted the therapeutic potential of GIPR agonism, with dual incretin receptor agonists demonstrating enhanced glycaemic control and weight loss in patients with T2D (Frias et al., 2018). The mechanisms underlying the enhanced weight loss exhibited by GIPR/GLP-1R co-agonism are yet to be fully characterised. Recent reports have highlighted the ability of GIPR agonism to attenuate nausea, emesis, and aversion associated with GLP-1RA treatment (Borner et al., 2021; Costa et al., 2022). In preclinical models GIPR / GLP-1R co-agonism has synergistic effects on appetite suppression (Coskun et al., 2018), suggesting a central mechanism of action regulating appetite and feeding behaviour.

*Gipr* and *Glp1r* are expressed in cell populations in the hypothalamus in both rodents and humans (Adriaenssens et al., 2019). In rodents, central expression of *Gipr* is necessary for the ability of GIPR/GLP-1R dual agonism to reduce body weight beyond that achieved by GLP-1R agonism alone, and chemogenetic activation of hypothalamic *Gipr* neurons acutely suppresses food intake (Adriaenssens et al., 2019; Zhang et al., 2021). Similarly, *Glp1r* expression in neurons of the CNS is necessary for GLP-1RA-mediated anorexia and body weight loss (Sisley et al., 2014). Blockade of GLP-1R in the arcuate nucleus of the hypothalamus attenuates the ability of a long-acting GLP-1RA to suppress food intake and body weight (Secher et al., 2014). Collectively these data demonstrate the potential importance of hypothalamic *Gipr*– and *Glp1r*–expressing cell populations to the regulation of pathways that control feeding and energy balance.

Recent advances in single-cell transcriptomics have enhanced our understanding of the heterogeneity of brain cells acting within molecularly-defined neurocircuits as well as non-neuronal cells that contribute to brain tissue maintenance and function. In this report, to better characterise hypothalamic *Gipr* and *Glp1r* populations and understand their relative similarities and differences, we used a transgenic approach combined with fluorescence activated cell sorting (FACS) to purify *Gipr*- and *Glp1r*- expressing hypothalamic cells and perform single-cell RNA sequencing (scRNAseq). Through downstream bioinformatic analysis, we have detailed and compared the composition and expression profiles of *Gipr*- and *Glp1r*- expressing hypothalamic cell populations, providing an in-depth analysis of both non-neuronal and neuronal cell types expressing these incretin receptors.

## 2 Methods

### 2.1 Animals

All animal procedures were approved by the University of Cambridge Animal Welfare and Ethical Review Body and conformed to the Animals (Scientific Procedures) Act 1986 Amendment Regulations (SI 2012/3039). The work was performed under the UK Home Office Project License PE50F6065. All mice were group-housed and maintained under SPF health/immune status in individually ventilated cages with standard bedding and enrichment unless otherwise stated. Mice were housed in a temperature (22°C) and humidity-controlled room on a 12 h light/dark cycle (lights on 7:00, lights out 19:00) with ad libitum access to water and standard laboratory chow diet (13.3% calories from fat, 22.4 % calories from protein, 64.3% calories from carbohydrate, 3.5 kcal/g; Scientific Animal Food Engineering).

*Gipr*-Cre (Adriaenssens et al., 2019) or *Glp1r*-Cre (Richards et al., 2014) mice were crossed with a *ROSA26*-EYFP reporter strain to enable fluorescent detection of cells expressing *Gipr* or *Glp1r*, producing *Gipr*^EYFP^ or *Glp1r*^EYFP^ mice, respectively. Cre lines and reporter strains were on a mixed C57B6J/N genetic background.

### 2.2 Flow Cytometry

Single cell suspensions were prepared from hypothalamic tissue pooled from two to six *Gipr*^EYFP^ or *Glp1r*^EYFP^ mice that were four to six weeks old as described previously (Adriaenssens et al., 2019). Both male and female mice were used. Briefly, mice were sacrificed by cervical dislocation, and tissue from the hypothalamus located ventrally caudal of the optical nerve chiasm (^~^Bregma -0.3 to -2.92 mm) was dissected into Hibernate-A medium without calcium (BrainBits). The tissue was digested with 20 U/ml Papain (Worthington) for 30 min at 37°C, followed by trituration in Hibernate-A medium (Thermo Fisher Scientific) containing 0.005% (w/v) DNase 1 (Worthington). The cell suspension was filtered through a 40 μm cell strainer into a fresh tube.

Fluorescence-activated cell sorting was performed using a BD Influx Cell Sorter (BD Biosciences, Franklin Lakes, NJ, USA) to isolate *Gipr*^EYFP+^ and *Glp1r*^EYFP+^ cells prepared from *Gipr*^EYFP^ or *Glp1r*^EYFP^ mice, respectively. Cells were gated according to cell size (FSC), cell granularity (SSC), FSC pulse-width for singlets, fluorescence at 488 nm/532 nm for EYFP and 647/670 nm for nuclear stain with DraQ5 (Biostatus).

### 2.3 Single Cell RNA Sequencing

Single cell cDNA libraries from purified *Gipr*^EYFP+^ or *Glp1r*^EYFP+^ cells were generated using the 10× Genomics Chromium Instrument and single-cell 3’ Reagent kit(V2 or V3 ; 10X Genomics). Pooled libraries were sequenced on an Illumina NovaSeq 6000 instrument (28-bp first read, 91-bp second read). Library preparation and sequencing was performed by the Genomics Core, Cancer Research UK Cambridge Institute. For downstream analysis, previously published sequencing data from hypothalamic *Gipr*^EYFP+^ cells (Adriaenssens et al., 2019) were included to increase the *Gipr*^EYFP+^ sample size.

Sequencing reads were aligned to an amended annotation of the mouse genome (GRCm39). The 3’ untranslated regions (UTRs) of *Glp1r*, *Ghsr*, *Prokr2* and *Prlhr* were compared against PolyA_DB v3 (Wang et al., 2018), and extended where necessary to include the furthermost 3’ UTRs in keeping with previous studies (Ludwig et al., 2021; Zhang et al., 2021). Downstream analyses on the unfiltered count matrices were performed using the Seurat v4 R package (Hao et al., 2021). Each sample was analysed individually prior to integration. Cells were filtered from the analysis if they expressed fewer than 500 unique genes or their total number of reads originating from mitochondrial genes was greater than 20%. There were a total of 14,091 cells in the filtered dataset, consisting of 1,614 and 1,210 from the two female *Gipr*^EYFP^ datasets, 8,527 from the male *Gipr*^EYFP^ dataset, and 2,740 from the male *Glp1r*^EYFP^ dataset.

Dimensionality reduction was performed on SCTransform (SCT)-normalised data, regressed for percentage of mitochondrial reads, percentage of ribosomal reads, and percentage of *Xist* reads. Uniform manifold approximation and projection (UMAP) were generated using the top 30 principle components from principle component analysis (PCA), run on variable features detected by SCT. Clustering was performed using the Louvain algorithm with resolution 0.8-1.0, and the k-parameter (k=15-30) for neighbour detection was reduced to the point where the smallest cluster visible was detected. Gene markers for each cluster were cross referenced against other bulk and single cell RNA sequencing databases RNAseq and in situ hybridisation (ISH) databases to assign cell type identities for each cluster (Campbell et al., 2017; Chen et al., 2017; Marques et al., 2016; Lein et al., 2007; Mickelsen et al., 2019; Mickelsen et al., 2020; Moffitt et al., 2018; He et al., 2016; Zeisel et al., 2018).

For individual cell type analysis, cells were filtered based on gene counts. Cells were selected using the following logic expressions: for vascular cells: (*Pdgfra* > 1 | *Pdgfrb* > 2 | *Cldn5* > 1 | *Tagln* > 3) & (*Mbp* < 1 | *Snap25* < 1); for oligodendrocytes, (*Olig1* > 2 | *Sox10* > 1 | *Mog* > 2) & (*Aqp4* < 1); for neurons, (*Syt1* > 1 | *Snap25* > 2) & (*Mustn1* < 5 | *Acta2* < 300 | *Mal* < 5). Vascular cells were further subdivided into pericytes, smooth muscle cells (SMCs), endothelial cells (ECs), and vascular leptomeningal cells (VLMCs) based on the expression of known markers from previous studies (*Kcnj8*, *Acta2*, *Cldn5*, and *Lum*, respectively) (Campbell et al., 2017; He et al., 2016; Zeisel et al., 2018). Filtered cells were re-analysed for dimensionality reduction per individual cell type, as described above.

Where differential expression analysis was performed, marker genes were identified for all clusters using a negative binomial regression model, implemented by Seurat’s FindAllMarkers function. Datasets were integrated using canonical correlation analysis (CCA), approximate nearest neighbours (ANN), and n = 2,000 integration features.

## 3 Results

### 3.1 Gipr and Glp1r expressing populations of the hypothalamus are heterogeneous

While multiple reports have used scRNAseq to survey and characterise neurons within the hypothalamus, *Gipr+* and *Glp1r+* populations represented in these studies are low in number, likely owing to difficulties in detecting lowly expressed transcripts at the sequencing depth afforded by single-cell and single-nucleus sequencing approaches. We therefore used *Gipr*-Cre and *Glp1r*-Cre transgenic mice crossed with a *ROSA26*-EYFP reporter strain to produce *Gipr*^EYFP^ and *Glp1r*^EYFP^ mice, allowing for the purification and enrichment of *Gipr*^EYFP+^ and *Glp1r*^EYFP+^ cells based on EYFP expression for detailed transcriptomic characterisation of these hypothalamic cell types.

Single-cell preparations isolated from hypothalamic samples of *Gipr*^EYFP^ or *Glp1r*^EYFP^ mice were FACS purified and encapsulated to create sequencing libraries for *Gipr*^EYFP+^ and *Glp1r*^EYFP+^ cells, respectively. We integrated *Gipr*^EYFP+^-and *Glp1r*^EYFP+^ data with a previously-published *Gipr*^EYFP+^ dataset to increase power for comparative analysis. Three sequencing libraries for *Gipr*^EYFP+^ cells and one sequencing library for *Glp1r*^EYFP+^ cells yielded a total of 11,351 *Gipr*^EYFP+^ cells and 2,740 *Glp1r*^EYFP+^cells for subsequent scRNAseq analysis (Supp. Fig. 1A).

**Figure 1:**
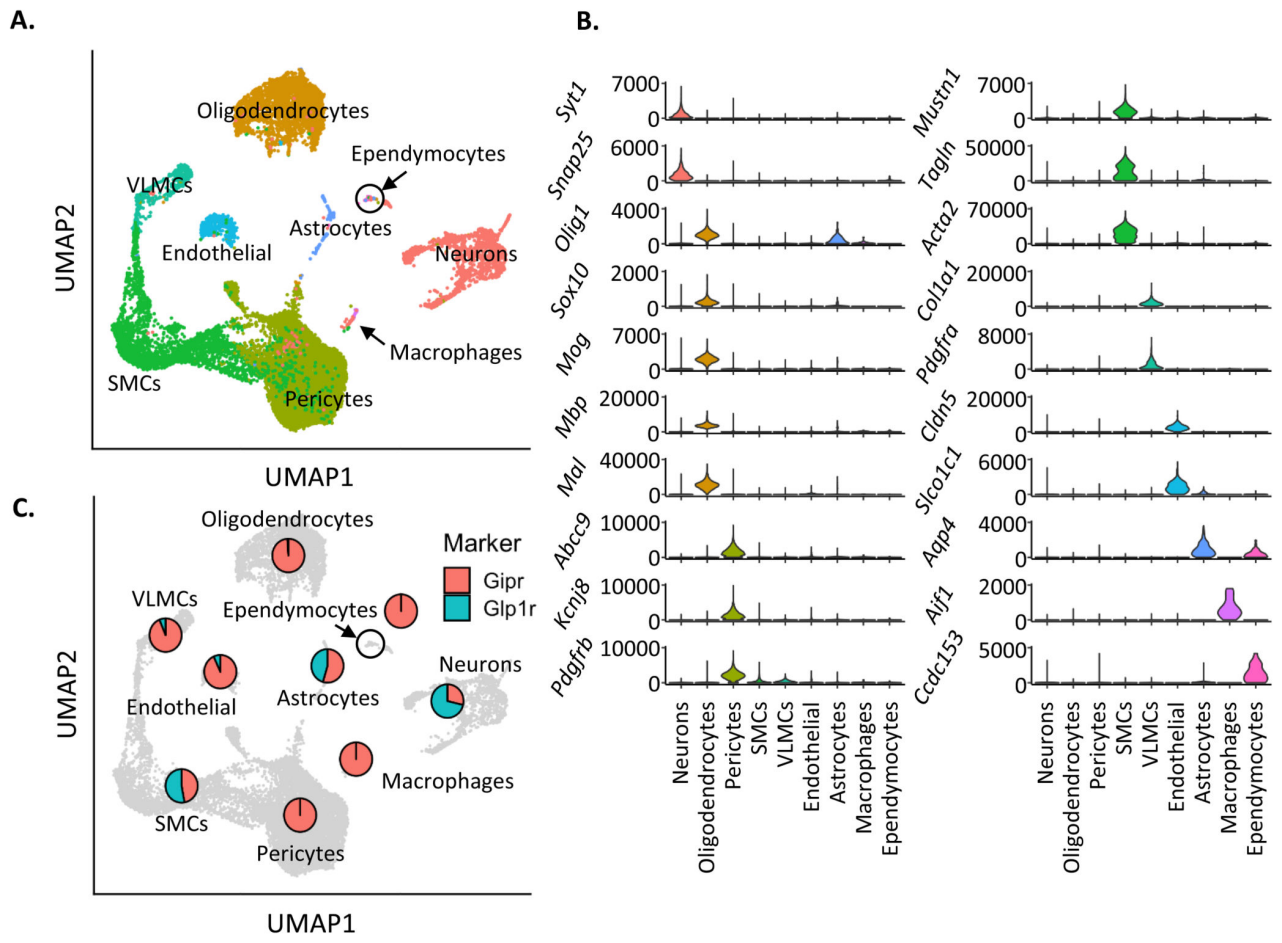
Transcriptomic characterisation of *Gipr*^EYFP+^ and *Glp1r*^EYFP+^ hypothalamic cells. (A) UMAP of the integrated *Gipr^EYFP^* and *Glp1r*^EYFP^ datasets, labelled for cell types identified during clustering of individual datasets. (B) Violin plots of marker gene expression per labelled cell type, plotting in counts per million (CPM). (C) UMAP of the integrated dataset, overlaid with pie charts representing proportion of *Gipr*^EYFP^ (red) and *Glp1r*^EYFP^ (teal) cells present in each cell type.

Unsupervised clustering analyses stratified these cells into 9 different cell type groupings (Fig 1 A). Cell type identities were assigned based on the expression of canonical marker genes (Figure 1 B Supp. Fig. 1A), indicating that both *Gipr*^EYFP+^and *Glp1r*^EYFP+^ populations are highly heterogeneous, with both neuronal and non-neuronal cell types represented (Campbell et al., 2017; He et al., 2016; Ludwig et al., 2021; Marques et al., 2016).

Among the detected cell types, neurons, oligodendrocytes (ODs), and multiple vascular cell types were identified within the *Gipr*^EYFP+^or *Glp1r*^EYFP+^ cell populations. Vascular cells, including pericytes, smooth muscle (SMCs), vascular leptomeningeal (VLMCs), and endothelial cells (ECs), formed the majority of the detected cells 70% (9879), while neurons and oligodendrocytes (ODs) accounted for 11% (1,526) and 17% (2,443) of the remaining cells, respectively. A small number of astrocytes (174), macrophages (26) and ependymocytes (43) were also found.

We explored the number of cells expressing *Gipr* and *Glp1r* transcript in each cell type cluster (Supplementary Fig 2A-C). Cells containing *Gipr* transcript mapped to all clusters except ependymocytes and macrophages, while cells containing *Glp1r* transcript mapped to all clusters except VLMCs. That not all cells contain *Gipr* or *Glp1r* transcript is expected given the difficulty in detecting low-expressing gene transcripts in scRNAseq data. In contrast, expression of Cre-dependent *EYFP* showed extensive coverage in the majority of cells in all clusters (Supplementary Fig 2D).

**Figure 2:**
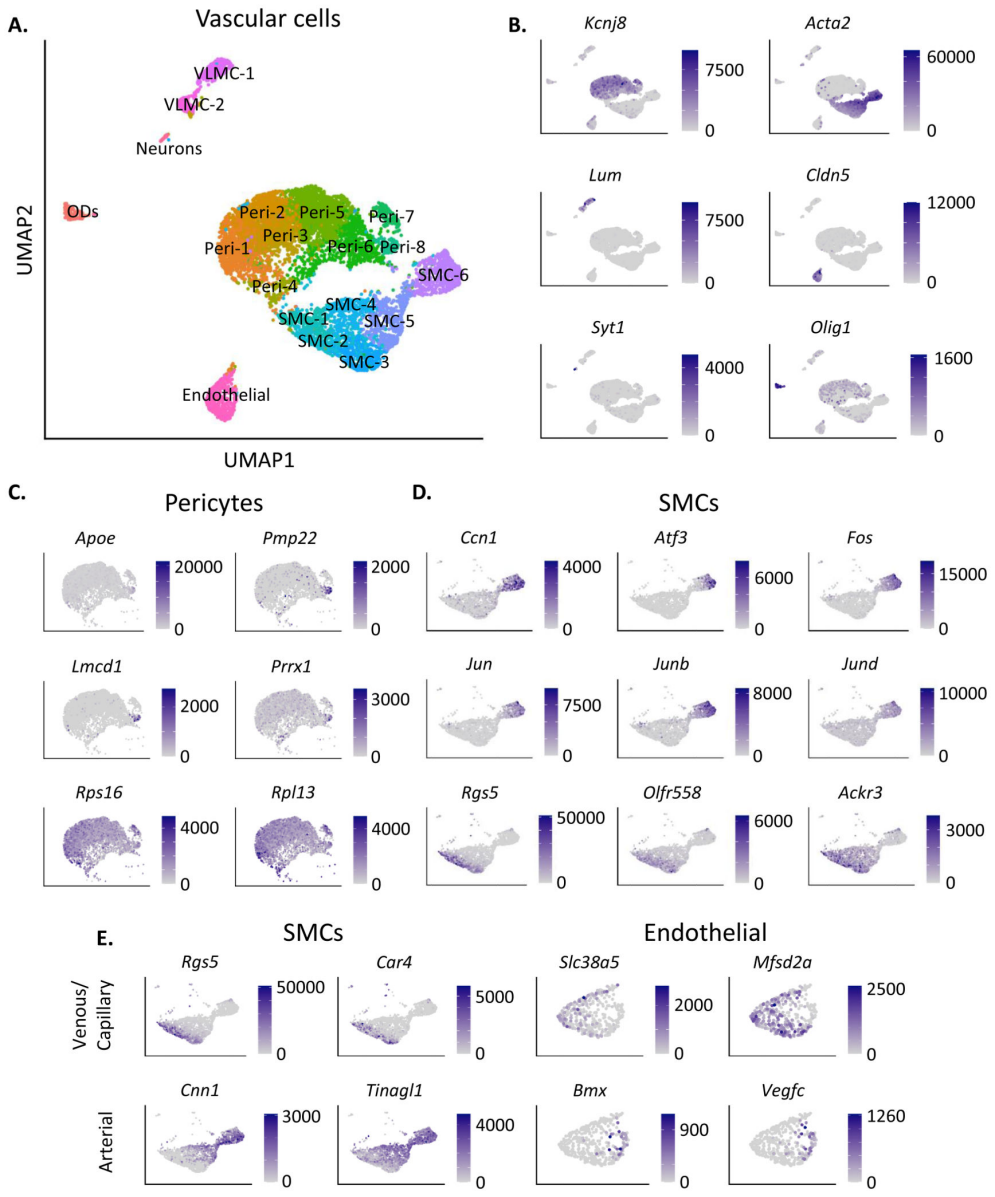
Comparative analysis of *Gipr*^EYFP+^ and *Glp1r*^EYFP+^ vascular cells. (A) UMAP of the vascular cells, selected using known cell type marker genes. (B) Gene expression of each cell type represented in the UMAP, including pericytes (*Kcnj8*), smooth muscle cells (SMCs; *Acta2*), vascular leptomeningeal cells (VLMCs; *Lum*), endothelial cells (*Cldn5*), neurons (*Syt1*) and oligodendrocytes (ODs; *Olig1*). (C) Gene expression of significantly differentially expressed (DE) genes in cluster Peri-8 compared to all other pericyte clusters (*Apoe*, *Pmp22*, *Lmcd1*, *Prrx1*), and DE genes between Peri-1,4 compared to Peri-5,6 (*Rps16*, *Rpl13*). (D) Gene expression of significantly differentially expressed (DE) genes in cluster SMC-6 (*Ccn1*, *Atf3*, *Fos*, *Jun*, *Junb*, *Jund*), and DE genes between SMC-1,2,3 compared to SMC-6. (E) Gene expression of marker genes for venous SMCs (*Rgs5*, *Car4*), arterial SMCs (*Cnn1*, *Tinagl1*), venous endothelial cells (*Scl38a5*, *Mfsd2a*), and arterial endothelial cells (*Bmx*, *Vegfc*). Gene expression is plotted in counts per million (CPM) in all plots.

To investigate the proportion of *Gipr*^EYFP+^ or *Glp1r*^EYFP+^cells represented by each cell type, the percentage of cells originating from the *Gipr*^EYFP+^or *Glp1r*^EYFP+^datasets within each cell type was calculated (Figure 1C). The *Glp1r*^EYFP+^dataset accounted for the majority of neurons, while pericytes were only found in the *Gipr*^EYFP+^datasets. About equal numbers of SMCs were detected from *Gipr*^EYFP+^or *Glp1r*^EYFP+^ datasets, and the *Gipr*^EYFP+^datasets accounted for the majority of both ODs and VLMCs. Considering that the *Gipr*^EYFP+^datasets account for more than 4x the number of cells compared to *Glp1r*^EYFP+^ in the integrated dataset, adjusting for sample sizes suggests that neurons and SMCs may be more heavily dominated by *Glp1r*^EYFP+^cells (Supplementary. Fig. 2E).

### 3.2 Vascular cells

Vascular cells were investigated by filtering for positive expression of gene markers for multiple known vascular cell types, including markers for pericytes (*Pdgfrb*), smooth muscle cells (SMCs) (*Tagln*), vascular leptomeningal cells (VLMCs) (*Pdgfra*), and endothelial cells (ECs) (*Cldn5*). Neurons and ODs were excluded using markers *Snap25* and *Mbp*, respectively. Dimensionality reduction for the 8,512 vascular cells reproduced the separation of pericytes, SMCs, VLMCs and ECs based on known cell type marker expression (pericytes: *Kcnj8*, SMCs: *Acta2*, ECs: *Cldn5*, VLMCs: *Lum*) (Figure 2A,B) (Campbell et al., 2017; He et al., 2016; Vanlandewijck et al., 2018; Zeisel et al., 2018). Dimensionality reduction of the vascular cell subset yielded 19 clusters separating into pericytes (Peri-1 to Peri-8), SMCs (SMC-1 to SMC-6), VLMCs (VLMC-1 to VLMC-2), and one cluster of ECs (Figure 2A). Small numbers of Neurons and ODs were seen in two peripheral clusters (Figure 2A).

Pericytes, demarcated by *Kcnj8* expression, formed eight clusters (Peri-1 to Peri-8) (Figure 2A). Significant differentially expressed genes driving the separation of cluster Peri-8 included the apolipoprotein *Apoe*, peripheral myelin protein *Pmp22*, and transcription factors *Lmcd1* and *Prrx1* (Figure 2C). Gene expression within clusters Peri-1, -2, -3, -4, -5, and -6 was relatively homogeneous, the only significant gene expression differences being higher levels of ribosome-related genes (e.g. *Rps16*, *Rpl13*) in cluster Peri-1 and Peri-4 (Fig 2C). Despite regression of sex-specific gene *Xist*, prior to clustering, *Xist* was the primary driver for separation of cluster Peri-7 (Supp. Fig. 3A).

**Figure 3:**
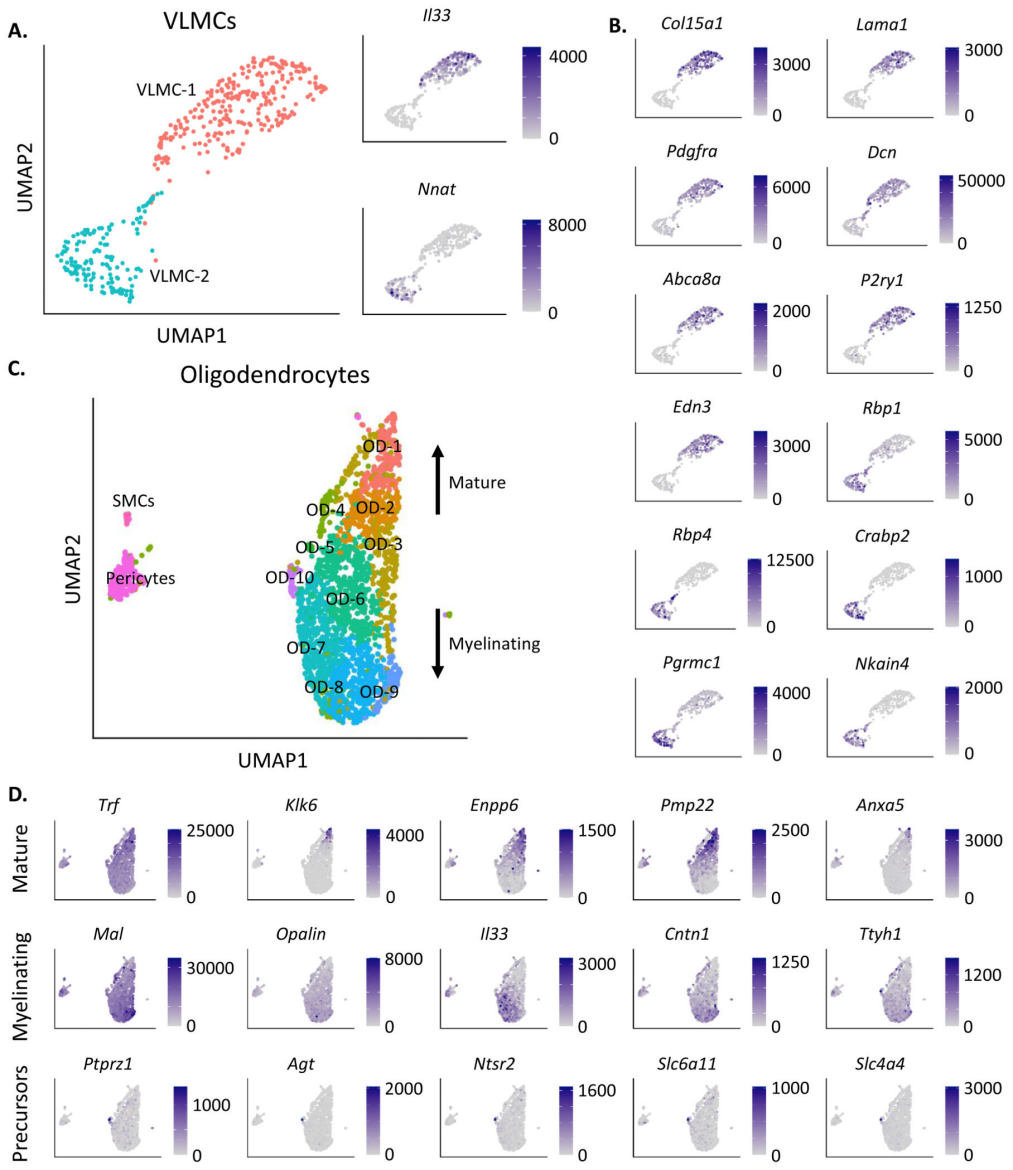
Transcriptomic characterisation of *Gipr*^EYFP+^ and *Glp1r*^EYFP+^ VLMC and oligodentroctye cells. (A) On the left, a UMAP of the VLMCs, labelled for the two detected clusters: VLMC-1 and VLMC-2. On the right, gene expression of two known markers for different types of VLMC, *Il33* and *Nnat*. (B) Gene expression of significantly differentially expressed (DE) genes enriched in cluster VLMC-1 (*Col15a1*, *Lama1*, *Pdgfra*, *Dcn*, *Abca8a*, *P2ry1*, *Edn3*) or VLMC-2 (*Rbp1*, *Rbp4*, *Crabp2*, *Pgrmc1*, *Nkain4*) relative to one another. (C) UMAP of oligodendrocytes (ODs), selected using known OD marker genes. Arrows annotate that OD maturity increased upwards from OD-9 to OD-1. (D) Gene expression of significantly differentially expressed (DE) genes enriched in OD-1,2 compared to OD-7,8,9 (*Trf*, *Klk6*, *Enpp6*, *Pmp22*, *Anxa5*), enriched in OD-7,8,9 compared to OD-1,2 (*Mal*, *Opalin*, *Il33*, *Cntn1*, *Ttyh1*), and DE genes in cluster OD-10 compared to all other OD clusters (*Ptprz1*, *Agt*, *Ntsr2*, *Slc6a11*, *Slc4a4*). Gene expression is plotted in counts per million (CPM) in all plots.

Of the six SMC clusters, identified by *Acta2* expression, SMC-6 expressed significantly higher levels of cell adhesion and proliferation gene *Ccn1*, and higher levels of transcription factors *Atf3*, *Fos*, *Jun*, *Junb* and *Jund* (Figure 2D). Each of these transcription factors have previously been shown to be enriched in arterial versus venous SMCs (Vanlandewijck et al., 2018). A subset of cells in SMC-1, -2, and-3 expressed higher levels of *Rgs5*, a marker for venous mural cells, as well as olfactory receptor *Olfr558*, and chemokine receptor *Ackr3* (Figure 2D). *Rgs5* was significantly enriched in the *Gipr*^EYFP+^ cells versus *Glp1r^EYFP+^* in SMC-5 and SMC-6 (Supplementary. Fig. 3B). *Olfr558* was significantly enriched in the *Gipr*^EYFP+^ cells versus *Glp1rEYFP+* in SMC-5 (Supplementary Fig 3B). These results suggest separation within the SMCs based on arteriovenous zonation, supported by the observation that venous SMC marker *Car4* was significantly enriched in the *Gipr*^EYFP+^ cells versus *Glp1r*^EYFP+^ in SMC-5, and arterial SMC markers *Cnn1* and *Tinagl1* were both enriched in *Glp1r*^EYFP+^ cells versus *Gipr*^EYFP+^ cells in SMC-6. *Tinagl1* was further enriched in *Glp1r*^EYFP+^ cells versus *Gipr*^EYFP+^ cells in SMC-1, -2, -4, -5 (Supplementary Figure 3C).

ECs, identified via *Cldn5* expression, were observed in a single cluster (Figure 2A), however investigation of arterial and venous endothelial gene markers suggested separation based on arteriovenous zonation within this cluster (Figure 2E). Cells stratified within the cluster based on differential enrichment of arterial endothelial markers (*Bmx* and *Vegfc*) and venous and capillary markers (*Slc38a5* and *Mfsd2a*) (Figure 2E). Though not significant, the arterial markers trended towards higher expression levels in *Glp1r*^EYFP+^ cells, while venous and capillary marker expression appeared higher in the *Gipr*^EYFP+^ cells (Supplementary Fig. 3D).

**Figure 4:**
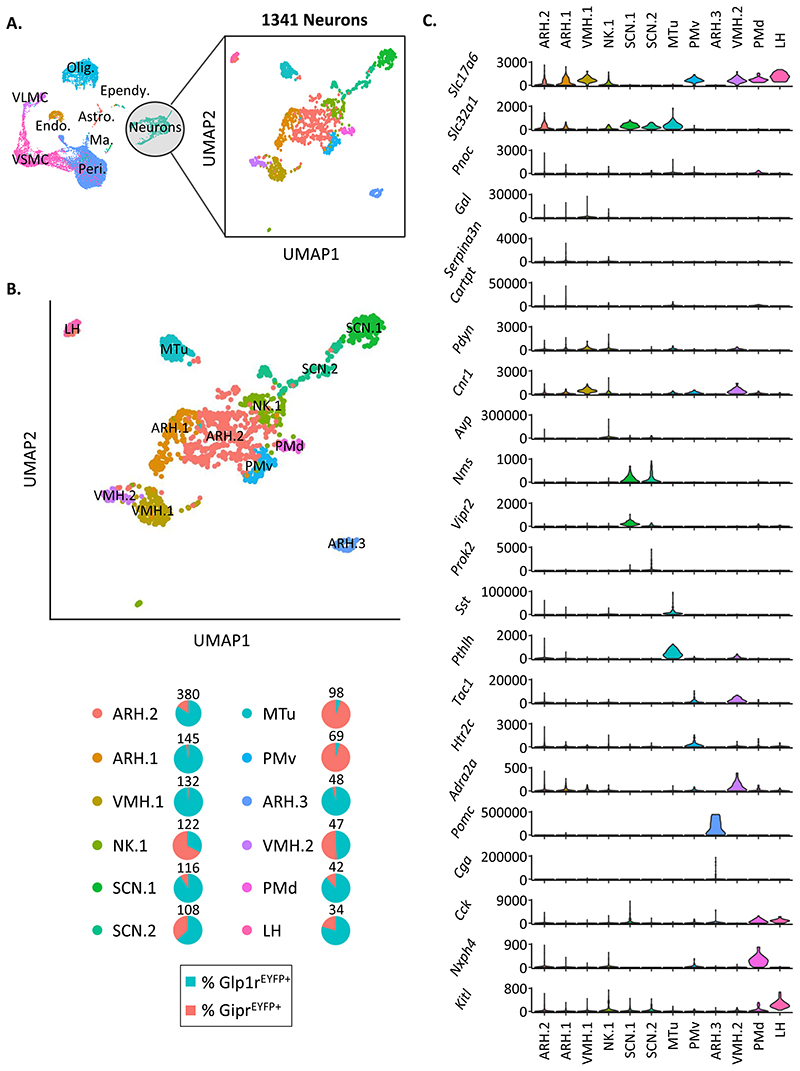
Characterisation of *Gipr*^EYFP+^ and *Glp1r*^EYFP+^ neurons. A) Neurons were subsetted based on positive expression of *Syt1* and *Snap25*. Cells expressing high levels of *Mustn1*, *Acta2*, or *Mal* were excluded, yielding 1341 *Gipr*^EYFP+^ and *Glp1r*^EYFP+^ neurons for downstream analysis. B) UMAP showing 12 separate clusters of *Gipr*^EYFP+^ and *Glp1r*^EYFP+^ neurons following dimensionality reduction and unsupervised clustering. Cluster-specific markers were identified using negative binomial regression analysis (see Table 1). The top 15 cluster-specific markers were cross-referenced with published brain region-specific transcriptional markers and the Allan Brain Atlas for region-specific mapping of clusters (see Supplementary Fig 5). Percentages of neurons from either *Gipr*^EYFP+^ or *Glp1r*^EYFP+^ neurons for each cluster are represented in pie charts. Total cell number per cluster is expressed at the top of each pie chart. C. Violin plots of neurotransmitters, secreted products, and cell surface receptors enriched in each neuronal cluster as determined by negative binomial regression analysis. Data are plotted in counts per million (CPM). ARH: arcuate hypothalamic nucleus, VMH: ventromedial hypothalamic nucleus, SCN: suprachiasmatic nucleus, LH: lateral hypothalamic nucleus, PM: premammillary nucleus, MTu: medial tuberal nucleus.

The VLMCs, distinguished by *Lum* expression, separated into two clusters (Figure 3A). There was no difference the *Gipr*^EYFP+^ versus *Glp1r*^EYFP+^composition in either VLMC cluster (Supplementary Fig. 4A). Zeisel *et al*. observed stratification of VLMCs in the adult mouse brain based on expression of the pro-inflammatory cytokine *Il33*, the prostaglandin D2 synthase *Ptgds* and neuronatin, *Nnat* (Zeisel et al., 2018). Cluster VLMC-1 expressed significantly higher levels of *Il33*, while cluster VLMC-2 expressed significantly higher levels of *Nnat* (Figure 3A). - *Ptgds* was not statistically differentially expressed between VLMC clusters (p_adj_ = 1) (Supplementary. Fig. 4B). *Nnat* and *Ptgds* expression was significantly enriched in *Gipr*^EYFP+^ versus *Glp1r*^EYFP+^ cells in VLMC-2 (Supplementary Fig. 4B). VLMC-1 was also found to express significantly higher levels of multiple VLMC/fibroblast-like markers, including *Col15a1*, *Lama1*, *Pdgfra* and *Dcn* (Figure 3B). Additional differentially expressed genes in VLMC-1 include ATP-binding cassette *Abca8a*, previously found to be correlated with expression of VLMC marker gene *Pdgfra* (Vanlandewijck et al., 2018), purinergic receptor P2Y *P2ry1*, and the endothelin *Edn3* (Figure 3B). VLMC-2 differentially expressed retinol binding proteins *Rbp1*, *Rbp4* and *Crabp2*, progesterone receptor *Pgrmc1*, and Na^+^ and K^+^ transporter *Nkain4* (Figure 3B). In VLMC-2 *Gipr*^EYFP+^ VLMC cells expressed significantly higher levels of *Pgrmc1* (Supplementary Fig. 4B).

### 3.3 Oligodendrocytes

To investigate *Gipr*^EYFP+^/ *Glp1r*^EYFP+^ODs further, cells were filtered for positive expression of general OD gene markers (*Olig1*, *Sox10*, *Mog*), and negatively against astrocytes (*Aqp4*). Filtering left 2,323 cells, which after re-clustering revealed 10 clusters of ODs (OD-1 to OD-10) (Figure 3C). Clusters OD-1 and OD-2 expressed higher levels of mature OD gene markers (*Trf*, *Klk6*), while clusters OD-7, OD-8 and OD-9 expressed higher levels of myelinating OD markers (*Mal*, *Opalin*) (Figure 3D). The mature OD clusters OD-1 and OD-2 also differentially expressed ectonucleotide metabolism gene *Enpp6*, peripheral myelin protein *Pmp22*, and annexin *Anxa5*. Myelinating OD clusters OD-7 and OD-8 expressed significantly higher levels of *Il33*, the contactin *Cntn1*, and the tweety family member *Ttyh1* (Figure 3D). OD-10, formed of 36 cells, had significantly higher expression of *Ptprz1*, a marker for OD precursors, as well as angiotensinogen *Agt*, neurotensin receptor *Ntsr2*, neurotransmitter transporter *Slc6a11*, and ion transporter *Slc4a4* (Figure 3D). The 32 *Glp1r*^EYFP+^cells were diffusely populated among all OD clusters (Supplementary Fig. 4C).

### 3.4 Neurons

To perform detailed analysis of the neurons represented in our data, cells were filtered for expression of *Syt1* and *Snap25*. Contaminating pericytes, SMCs, and ODs were eliminated based on expression of *Abcc9*, *Acta2*, and *Mal*, respectively. 397 *Gipr*^EYFP+^ and 944 *Glp1r*^EYFP+^ resultant neurons clustered into 12 distinct sub-populations following dimensionality reduction (Fig 4A).

The tissue preparation enabling scRNAseq results in a loss of the spatial context of neurons represented in our sample. To infer the anatomical distribution of *Gipr*^EYFP+^ and *Glp1r*^EYFP+^neuronal clusters to discrete hypothalamic nuclei, the top 15 cluster-enriched gene markers extracted from differential gene expression analysis were compared to available nucleus-specific scRNAseq datasets and were mapped to the Allan Brain Atlas (Campbell et al., 2017; Chen et al., 2017; Lein et al., 2007; Mickelsen et al., 2019; Mickelsen et al., 2020; Moffitt et al., 2018) (Supplementary Fig 5; Supplementary Table 1). Regional mapping revealed three clusters from the arcuate nucleus (ARH.1, ARH.2, ARH.3) two clusters from the ventromedial hypothalamic nucleus (VMH.1, VMH.2), two clusters from the suprachiasmatic nucleus (SCN.1, SCN.2), one cluster from the lateral hypothalamus (LH), two clusters from the premammillary nucleus (PMv, PMd), one cluster from the medial tuberal nucleus (MTu), and one cluster that remains unassigned (NK.1) (Fig 4B). Eight of the 12 clusters were predominantly composed of *Glp1r*^EYFP+^ neurons, 3 were predominantly represented by *Gipr*^EYFP+^ neurons, and one had equal representation of *Glp1r*^EYFP+^ and *Gipr*^EYFP+^ neurons (Fig 4B).

In addition to regional markers, the neuronal clusters were stratified by their relative enrichment for neurotransmitters and genes encoding secreted products and cell surface receptors (Fig 4C). All three ARH clusters were predominantly *Glp1r*^EYFP+^, with ARH.2 exhibiting the highest percentage of *Gipr*^EYFP+^ neurons (16%). Expression of *Slc17a6* (encoding a vesicular transporter for glutamate) and *Slc32a1* (encoding GABA vesicular transporter) indicated that ARH.1 and ARH.2 were both hybrid glutamateric/GABAergic cell populations. Enrichment of *Pnoc* (encoding prepronociceptin) expression distinguished ARH.1, with increased *Cartpt*/*Serpina3n* (encoding cocaine- and amphetamine-regulated transcript protein and the serine protease inhibitor A3N precursor, respectively) expression identifying ARH.2. ARH.3 was highly enriched for *Pomc* and *Cga*, (encoding proopiomelanocortin and the alpha subunit of the glycoprotein hormone family, respectively). Neurons in NK.1 were principally *Gipr*^EYFP+^, and express both *Slc17a6* and *Slc32a1*, with enriched expression of arginine vasopressin transcript (*Avp*).

VMH.1, VMH.2, LH, PMd, and PMv were all principally glutamatergic (*Slc17a6*+). VHM.2 was composed of equal proportion of *Gipr*^EYFP+^ and *Glp1r*^EYFP+^ neurons, whereas the majority of VMH.1 neurons are *Glp1r*^EYFP+^. VMH.1 is enriched for prodynorphin transcript (*Pdyn*) and the cannabinoid receptor transcript, Cnr1, with VMH.2 expressing high levels of tachykinin precursor, *Tac1* and the alpha-2-adrenergic receptor, *Adra2a*. Though not significantly enriched, the majority of neurons in the LH cluster expressed *Cck* (encoding cholecystokinin). The PMd cluster also expressed *Cck*, with a significant enrichment of neurexophilin4 (*Nxph4*). In contrast to PMd, PMv was principally *Gipr*^EYFP+^, and enriched for *Tac1* and the serotonin receptor, *Htr2c*.

The MTu, SCN.1, and SCN.2 clusters were GABAergic (*Scl32a1*+). Both SCN clusters were enriched for neuromedin S transcript (*Nms*). SCN.1 expressed higher levels of the vasoactive intestinal peptide receptor 2 (*Vipr2*), and demonstrated a relatively higher percentage of *Glp1r*^EYFP+^ neurons. MTu was 95% *Gipr*^EYFP+^, and was significantly enriched for somatostatin (*Sst*) and parathyroid hormone-like hormone (*Phtlh*) transcripts.

## 4 Discussion

Rich insight into the heterogeneity and identity of cells is afforded by single cell transcriptomic approaches, though limitations in sensitivity can be problematic for target cell populations identified by lowly expressed genes. Expression levels for *Gipr* and *Glp1r* are lower than for genes encoding neuropeptides and secreted hormones, presenting a barrier to mining existing transcriptomic datasets for cell types defined by either *Gipr* or *Glp1r* where their representation is either extremely low or not at single-cell resolution (Biglari et al., 2021; Lam et al., 2017). Through the use of transgenic mouse models, our approach enabled the purification and enrichment of *Gipr*^EYFP+^ and *Glp1r*^EYFP+^ populations, resulting in a dataset of over 14,000 cells.

While we have previously shown that *Gipr*^EYFP+^ cells of the hypothalamus are composed of both neuronal and non-neuronal cell types, our present analysis revels that *Glp1r*^EYFP+^ cells are similarly heterogeneous. In particular *Glp1r*^EYFP+^and *Gipr*^EYFP+^cells were found in a variety of vascular cell types. *Gipr*^EYFP+^cells localised to all observed vascular cell clusters, including pericytes, SMCs, VLMCs, and ECs. In contrast, *Glp1r*^EYFP+^ cells were found in VLMCs, ECs, SMCs, but not pericyte clusters. Pericytes have wide-ranging effects on the cerebrovasculature, from modulating endothelial transcytosis and the polarization of astrocyte end-feet, to regulating neuroinflammatory responses, cerebral blood flow and the integrity of the vascular basement membrane (Armulik et al., 2010; Hall et al., 2014; Török et al., 2021; Vanlandewijck et al., 2018). Additionally, gene ontology analysis has previously shown that genes encoding transmembrane transporters are organotypic for brain pericytes over pericyte cells isolated from peripheral regions, suggesting that pericytes of the brain are directly involved in molecular transport at the blood brain barrier (BBB) (Vanlandewijck et al., 2018). Whether acute manipulation of pericyte activity via GIPR engagement affects the transport and uptake of nutrients, peptides, and other regulatory factors from the periphery across the BBB should be investigated.

The absence of *Glp1r*^EYFP+^ pericytes is likely due to the arteriovenous zonation of pericytes, which are transcriptomically more similar to venous SMCs (Vanlandewijck et al., 2018). This is supported by the observation that the *Glp1r*^EYFP+^ SMCs express higher levels of arterial gene markers, and appear transcriptomically distinct from the *Gipr*^EYFP+^ SMCs, which instead express higher levels of venous gene markers. This is further supported by the increased expression of arterial endothelial cell markers in the *Glp1r*^EYFP+^vascular cells compared to *Gipr*^EYFP+^vascular cells. These data suggest that GLP-1 may signal principally through arterial cells whereas GIP may target venous cells of the cerebrovasculature.

*Glp1r* has previously been shown to be expressed in vascular smooth muscle cells of large and small arteries in the periphery (Richards et al., 2014). Systemic administration of GLP-1RAs has been demonstrated to protect brain tissue from ischaemia-induced damage, supporting a role for GLP-1R engagement in modulating perfusion of brain tissue (Hakon et al., 2015). While these protective effects of GLP-1RAs may be attributable to engagement of GLP-1R in the carotid body (Pauza et al., 2022), in acute brain slices, application of a short-acting GLP-1RA had a strong dilatory effect on cortical arterioles and GLP-1R agonism elicited increased cerebral blood flow *in vivo*, indicating that direct GLP-1R engagement at the level of the cerebrovasculature may also be important for regulating brain tissue perfusion and oxygenation (Nizari et al., 2021). Given that the majority of *Gipr*^EYFP+^ cells in the hypothalamus express markers for vascular cells, GIPR activation may also modulate cerebral blood flow. Though exogenous GIP infusion has previously been reported to increase blood flow in the periphery in adipose tissue (Asmar et al., 2016; Asmar et al., 2017), effects of GIPR agonism on brain perfusion are yet to be demonstrated.

All oligodendrocyte clusters in our data were predominantly composed of *Gipr*^EYFP+^ cells. Expression of *Gipr* transcript has been previously reported in oligodendrocytes from both the hypothalamus and the dorsal vagal complex (Dowsett et al., 2021; Kohnke et al., 2021). Oligodendrocytes are acutely sensitive to changes in nutritional signals, with fasting triggering their rapid proliferation and differentiation (Kohnke, S Cell Rep, 2021). In the hindbrain, oligodendrocyte remodelling in response to fasting was driven by changes in *Tcf7l2* expression (Dowsett et al., 2021). Whether GIPR agonism induces transcriptional changes and elicits cell differentiation in oligodendrocytes elicits is currently unknown and warrants further exploration.

The majority of oligodendrocytes in our data expressed markers indicative of mature and myelinating oligodendrocytes. It is important to note that the tissue samples from which our data were collected were isolated from young mice (four to six weeks in age), potentially before myelination is complete. Whether the relative representation of oligodendrocyte precursor cells verses mature oligodendrocytes expressing incretin receptors changes with maturity should be evaluated.

In this study we find that a small population of astrocytes express *Glp1r*. While these astrocytes demonstrate low *Gfap* expression, they instead express *Aqp4*, a universal marker for astrocytes encoding the water channel, aquaporin 4 that is located on astrocyte vascular end-feet (Zeisel et al., 2018). The fact that incretin receptor-expressing astrocytes are low in *Gfap* suggests that they are parenchymal protoplasmic astrocytes, rather than fibrous astrocytes known for their high levels of *Gfap* expression (Zeisel et al., 2018). A role for *Glp1r* in glial and astrocyte biology is supported by studies examining GLP-1R pharmacology in these cell types (Cui et al., 2022). Specifically, in calcium imaging studies one third of astrocytes in the dorsal vagal complex were responsive to GLP-1R agonism, and pharmacological blockade of astrocytes in the NTS attenuated GLP-1RA-dependent anorexia (Reiner et al., 2016). Due to their relative low abundance, it is likely that incretin receptor-expressing astrocytes work in conjunction with other *Gipr* and *Glp1r* expressing cell types to mediate changes in energy balance following pharmacological activation.

We were able to identify *Gipr*^EYFP+^and *Glp1r*^EYFP+^neuronal cells originating from discrete hypothalamic nuclei through mapping transcriptomic cluster signatures to ISH references. Localisation of Glp1r+ neurons to the ARH, VMH, LH, SCN and premammillary nuclei is in good agreement with previous immunohistochemical studies (Cork et al., 2015; Jensen et al., 2018). Of note, no *Gipr*^EYFP+^ or *Glp1r*^EYFP+^ clusters were mapped to the paraventricular hypothalamus (PVH) or dorsomedial hypothalamus (DMH). Previous immunohistochemistry studies indicate that *Gipr*+ and *Glp1r*+ neurons are present in these nuclei (Adriaenssens et al., 2019; Cork et al., 2015). The cluster, NK.1 may represent cells from the PVH or DMH, though the transcriptional markers for this cluster largely failed to specifically map this cluster. Our data build on previous immunohistochemical studies through Identifying key neuropeptide and cell surface identifiers for *Glp1r*^EYFP+^ and *Gipr*^EYFP+^ clusters, enriching our knowledge of factors that distinguish *Glp1r*- and *Gipr*-expressing cells residing in separate hypothalamic nuclei.

### 4.1 Limitations of Study

Our study relies on a transgenic approach where *Glp1r*- and *Gipr*-expressing cells have been labelled with an endogenous fluorescent reporter using *Glp1r*-Cre and *Gipr*-Cre mice, respectively. Transgenic Cre drivers can result in aberrant Cre expression or lineage tracing artefacts. Additionally, mRNA expression does not always mirror protein translation, which has been demonstrated for GLP-1R in pancreatic delta cells where *Glp1r* transcript was detected, but did not correlate with GLP-1R protein levels as measured using antibody staining (Gray et al., 2020). Recently, however Jensen *et al* described a specific GLP-1R monoclonal antibody (mAb) for immunohistochemical (IHC) analysis of mouse brain tissue. In this study the authors compared the distribution of GLP-1R mAb staining to a report by Cork *et al* where the *Glp1r*^EYFP^ mouse model we present here was used to map Cre-dependent EYFP expression in mouse brain (Cork et al., 2015; Jensen et al., 2018). Jensen *et al* reported strong correlation between GLP-1R monoclonal antibody staining with *Glp1r*-Cre-dependent EYFP staining. Importantly, no regions that were positive for *Glp1r*-Cre-dependent EYFP staining were found to be negative for GLP-1R mAb staining (Jensen et al., 2018). At present, no GIPR antibodies have been validated for use in IHC in whole tissue. The *Gipr*-Cre model used in this study is a knock-in Cre, which is less likely to result in aberrant Cre expression compared to transgenic models using randomly integrated constructs. While we cannot exclude that some cells may report Cre activity in the absence of active *Gipr* transcription, in our previous work we confirmed that *Gipr*-Cre-dependent EYFP expression in the hypothalamus corroborated the detection of *Gipr* mRNA using single molecule fluorescent *in situ* hybridization (Adriaenssens et al., 2019). Future studies should further confirm the anatomical phenotyping provided in this report using cell type-specific smFISH markers and functional assays measuring GIPR and GLP-1R activity.

The *Glp1r*-Cre model used in this report employs a strategy relying on the integration of a bacterial artificial chromosome (BAC) (Richards et al., 2014). Two additional transgenic *Glp1r*-Cre mouse lines have since been created, both relying on a knock-in approach to introduce Cre-recombinase expression under the control of the *Glp1r* promotor (Andersen et al., 2021; Williams et al., 2016). Discrepancy in peripheral cell types labelled by the knock-in versus the BAC approach has been reported, with pancreatic acinar cells labelled by a knock-in model whereas no exocrine pancreatic cells were labelled using the BAC model (Andersen et al., 2021; Richards et al., 2014). While this divergence may reflect the low expression level of *Glp1r* in exocrine tissue, ideally similar studies to those presented in this work should be performed using a *Glp1r*-Cre knock-in model to explore whether discordance exists at the transcript level in brain cells expressing *Glp1r*.

### 4.2 Summary

Here we provide transcriptomic molecular identification of neuronal and non-neuronal cells in the hypothalamus that express receptors for the incretin hormones, GIP and GLP-1. This census of cell types expressing *Glp1r*^EYFP+^and *Gipr*^EYFP+^ will be an important resource for understanding the molecular fingerprint of these cells, and provides insight for future functional studies.

## Supplementary Material

Supplementary Figures

## Data Availability

The data are available from the NCBI Gene Expression Omnibus accession GSE199301.

## Abbreviations

ARH: arcuate hypothalamic nucleus
VMH: ventromedial hypothalamic nucleus
SCN: suprachiasmatic nucleus
LH: lateral hypothalamic nucleus
PM: premammillary nucleus
MTu: medial tuberal nucleus
GLP-1: glucagon-like peptide-1
GLP-1R: Glucagon-like peptide-1 receptor
GIP: Glucose-dependent insulinotropic polypeptide
GIPR: Glucose-dependent insulinotropic polypeptide receptor
