# Supplementary material for: A comparative transcriptomic analysis of Glucagon-like peptide-1 receptor- and Glucose-dependent insulinotropic polypeptide receptor-expressing cells in the hypothalamus
Source: Appetite. Author manuscript; Available in PMC 2023 Mar 28. (PMC7614381; doi:10.1016/j.appet.2022.106022)
Supplement: Supplementary Figures [file EMS172276-supplement-Supplementary_Figures.pdf]

Supplementary Figure 1. Datasets included for transcriptomic analysis of *Gipr*$^{EYFP+}$ and *Glp1r*$^{EYFP+}$ hypothalamic cells (A) Cell type labelling of UMAPs per dataset (from left to right: *Gipr*^{EYFP} female 1, *Gipr*^{EYFP} female 2, *Gipr*^{EYFP} male, *Glp1r*^{EYFP} male). Violin plots of gene expression for marker genes per cell type detected in each dataset. Gene expression is plotted in counts per million (CPM).

Supplementary Figure 2: Feature plots highlighting cells expressing *Glp1r*, *Gipr*, or *EYFP*. A-C. Cells that express > 0 transcript counts for (A) *Gipr*, (B) *Glp1r*, (C) *EYFP* are highlighted in blue. D. UMAP plot designating cell type clusters. (E) UMAP of the integrated dataset, overlaid with pie charts representing size-adjusted proportion of *Gipr*^{EYFP} (red) and *Glp1r*^{EYFP} (teal) cells present in each cell type, based on increasing the contribution from *Glp1r*^{EYFP} by more than 4-fold (= 11,351/2,740).

Supplementary Figure 3. Differential expression analysis of *Gipr*$^{EYFP+}$ and *Glp1r*$^{EYFP+}$ SMC and endothelial cells. (A) Gene expression of *Xist* in vascular cluster Peri-7. (B) Gene expression of differentially expressed (DE) genes between clusters of smooth muscle cells (SMCs), split by dataset marker (*Gipr* or *Glp1r*). * = p-adj<0.05. (C) Gene expression of marker genes for venous (*Rgs5*, *Car4*), arterial (*Cnn1*, *Tinagl1*) and arteriole SMCs (*Cd93*, *Nanos1*), split by dataset marker (*Gipr* or *Glp1r*). * = p-adj<0.05. (D) UMAP of vascular cells, labelled for dataset. (E) Gene expression of marker genes for venous (*Bmx*, *Vegfc*, *Gkn3*) and arterial endothelial cells (*Mfsd2a*, *Ivns1abp*, *Slc16a1*), split by dataset marker (*Gipr* or *Glp1r*). * = p-adj<0.05. Gene expression plotted in counts per million (CPM) for all plots.

Supplementary Figure 4. Differential expression analysis of *Gipr*$^{EYFP+}$ and *Glp1r*$^{EYFP+}$ VLMC cells. (A) Gene expression of differentially expressed (DE) genes between clusters of VLMCs, split by dataset marker (*Gipr* or *Glp1r*). * = p-adj<0.05. Gene expression plotted in counts per million (CPM). (B) UMAP of the VLMCs, overlaid with pie charts representing proportion of *Gipr*^{EYFP} (red) and *Glp1r*^{EYFP} (teal) cells present in each cell type. (C) UMAP of oligodendrocytes, labelled for dataset marker (*Gipr* or *Glp1r*).

Supplementary Figure 5: Regional markers used for neuronal cluster identification. Cluster-specific markers were identified using negative binomial regression analysis (see Table 1), compared to published brain region-specific transcriptional markers, and mapped to specific hypothalamic nuclei using the Allan Brain Atlas. Feature plots show selected cluster marker expression are shown on left.

Data are expressed in CPM. Representative ISH images form the Allan Brain Atlas for each cluster are shown on right.

Supplementary Table 1: Top 15 Neuronal cluster markers. Cluster markers were determined using negative binomial regression analysis. The top 15 markers per neuronal cluster are listed.

Supp Fig 1

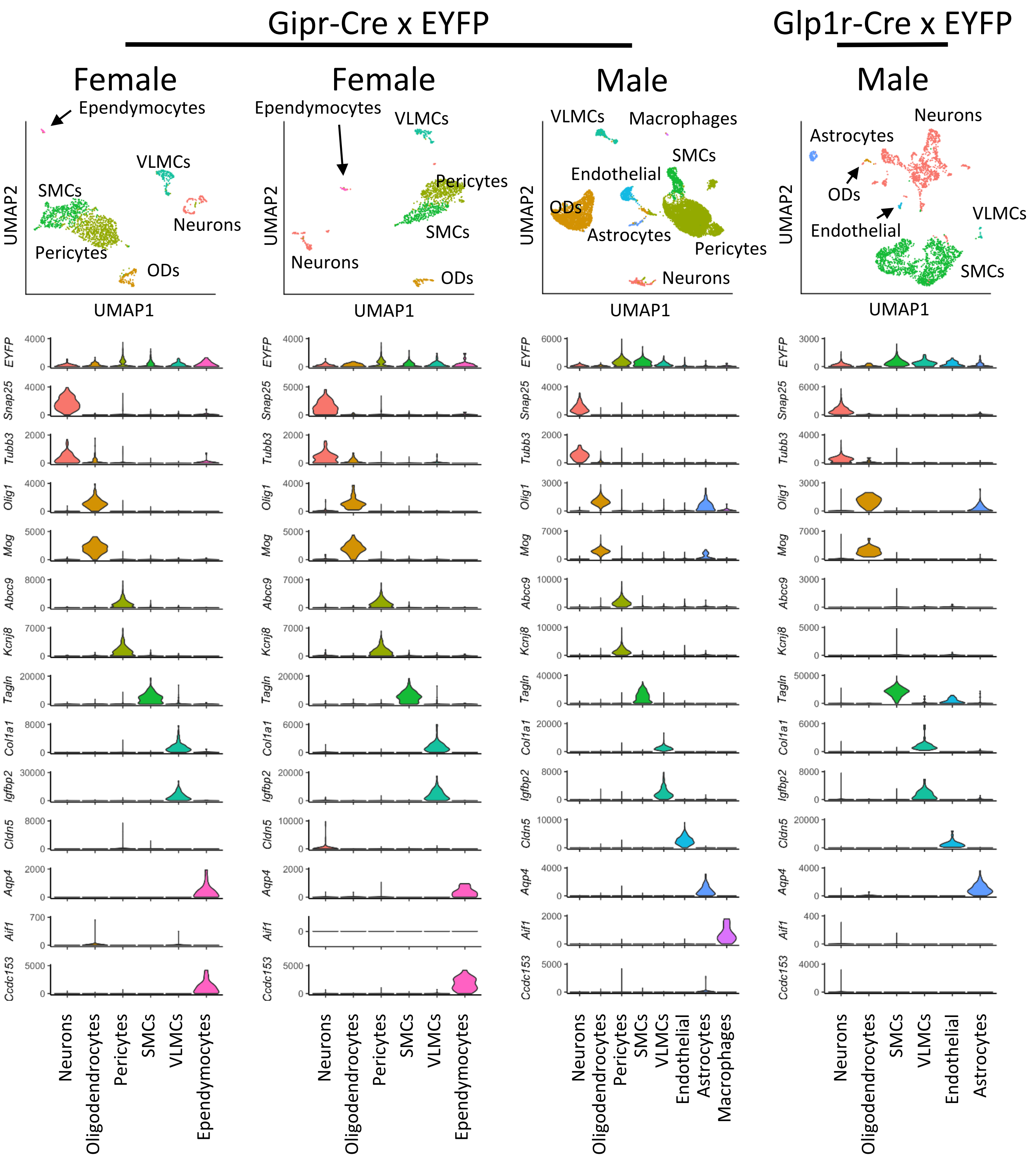
Gipr-Cre x EYFP
Glp1r-Cre x EYFP
Female
Female
Male
Male
Ependymocytes
VLMCs
SMCs
Neurons
Pericytes
ODs
Macrophages
Endothelial
Astrocytes
UMAP1
UMAP2
EYFP
Snap25
Tubb3
Olig1
Mog
Abcc9
Kcnj8
Tagln
Col1a1
Igfbp2
Cldn5
Aqp4
Aif1
Ccdc153
Neurons
Oligodendrocytes
Pericytes
SMCs
VLMCs
Ependymocytes
Endothelial
Astrocytes
Macrophages

Supp Fig 2

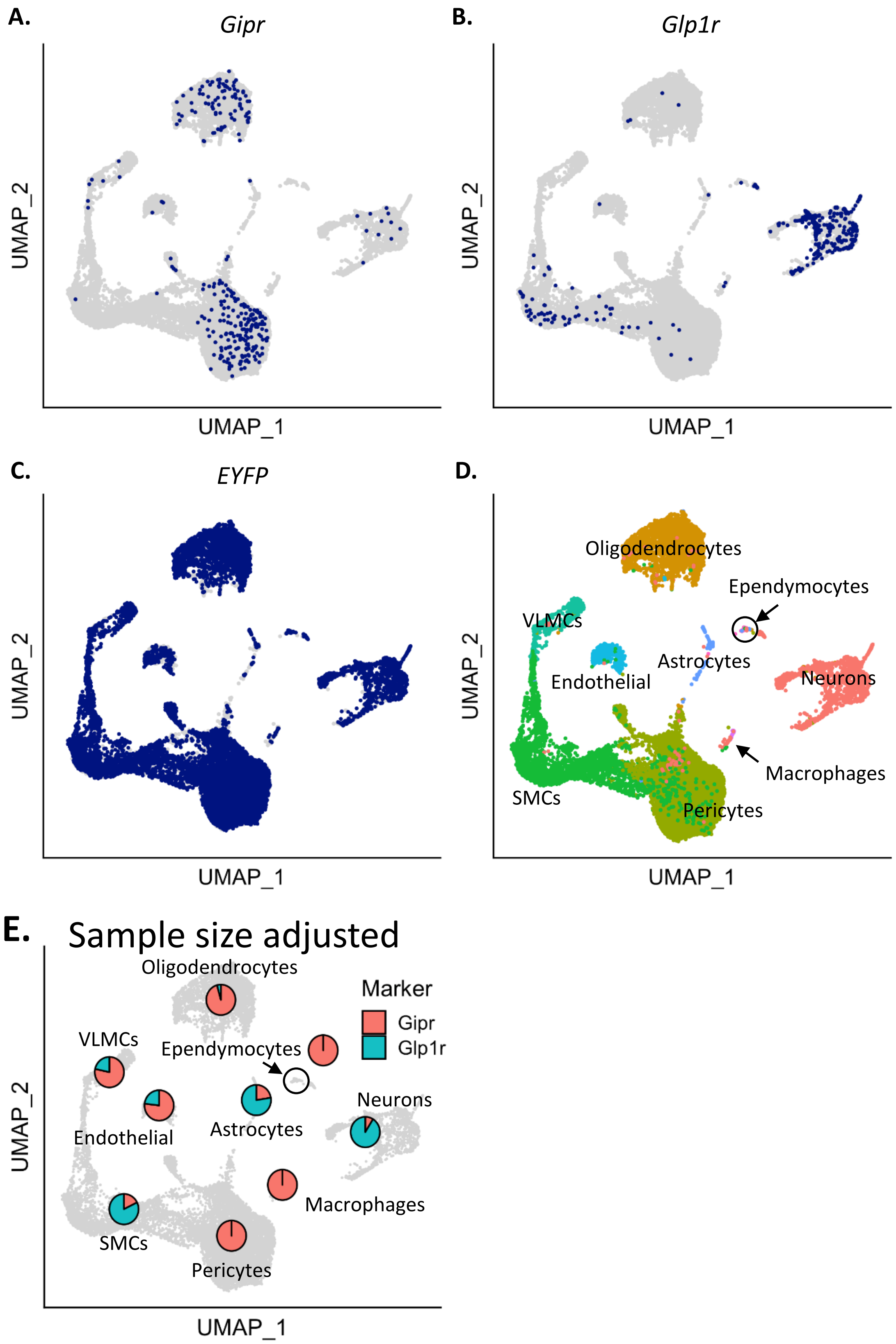
A.
Gipr
UMAP_2
UMAP_1
B.
Glp1r
UMAP_2
UMAP_1
C.
EYFP
UMAP_2
UMAP_1
D.
Oligodendrocytes
Ependymocytes
VLMCs
Astrocytes
Endothelial
Neurons
Macrophages
SMCs
Pericytes
UMAP_2
UMAP_1
E.
Sample size adjusted
Oligodendrocytes
Marker
Gipr
Glp1r
VLMCs
Ependymocytes
Neurons
Endothelial
Astrocytes
Macrophages
SMCs
Pericytes
UMAP_2
UMAP_1

Supp Fig 3

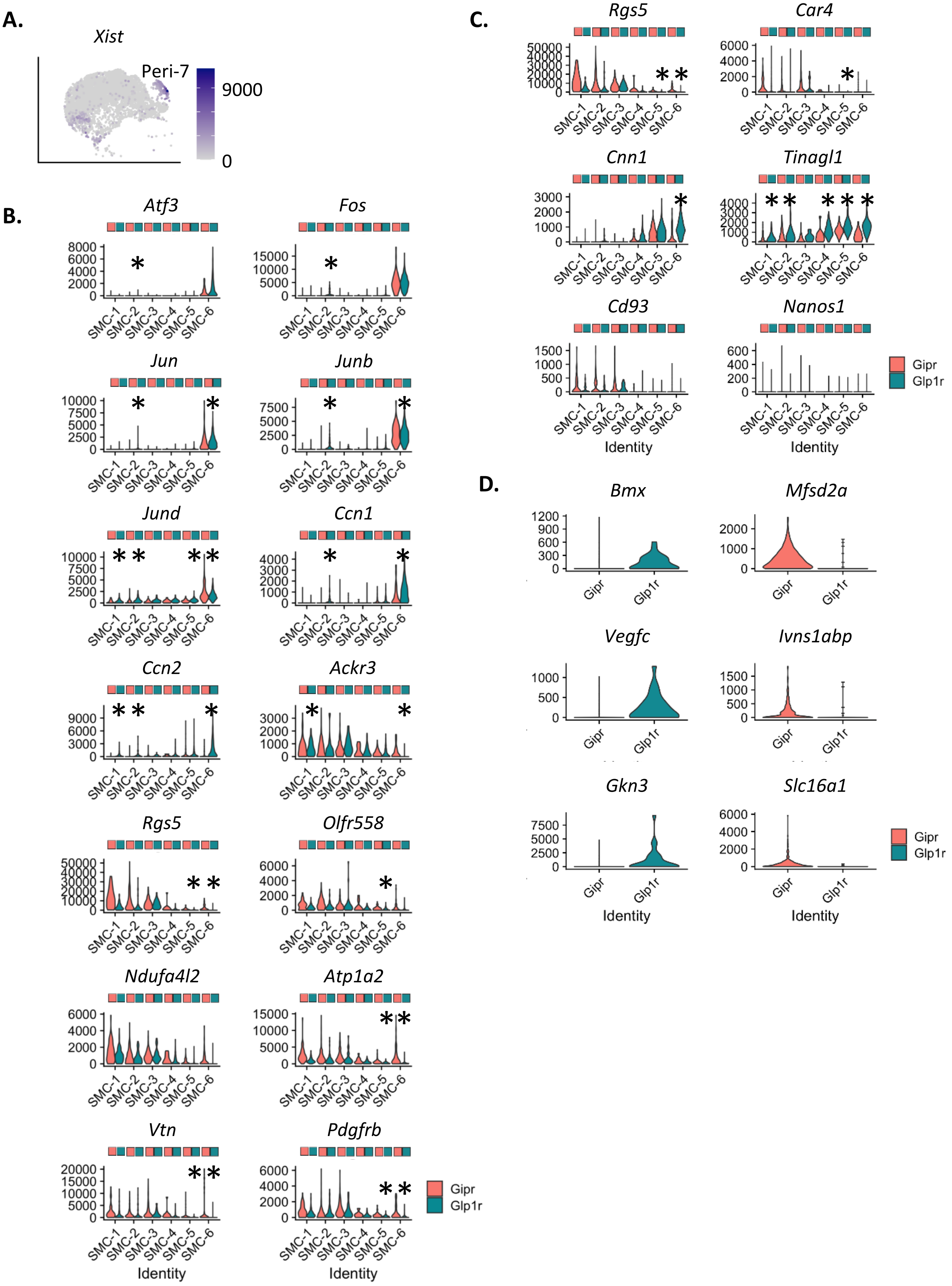
A.
Xist
Peri-7
9000
0
B.
Atf3
Fos
Jun
Junb
Jund
Ccn1
Ccn2
Ackr3
Rgs5
Olfr558
Ndufa4l2
Atp1a2
Vtn
Pdgfrb
SMC-1
SMC-2
SMC-3
SMC-4
SMC-5
SMC-6
Identity
Gipr
Glp1r
C.
Rgs5
Car4
Cnn1
Tinagl1
Cd93
Nanos1
Identity
Gipr
Glp1r
D.
Bmx
Mfsd2a
Vegfc
Ivns1abp
Gkn3
Slc16a1
Identity
Gipr
Glp1r

Supp Fig 4

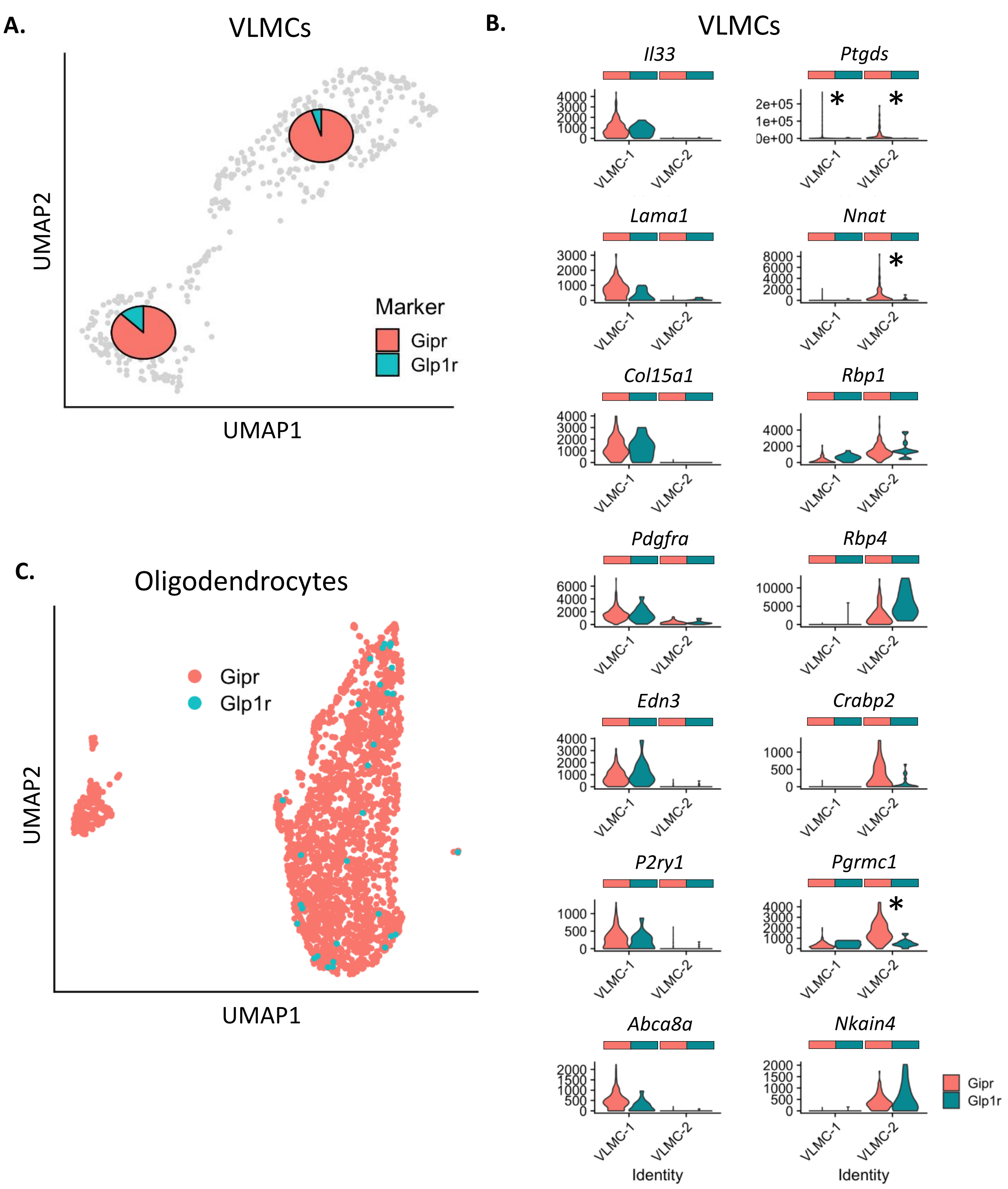
A.
VLMCs
UMAP2
UMAP1
Marker
Gipr
Glp1r
B.
VLMCs
Il33
Ptgds
Lama1
Nnat
Col15a1
Rbp1
Pdgfra
Rbp4
Edn3
Crabp2
P2ry1
Pgrmc1
Abca8a
Nkain4
VLMC-1
VLMC-2
Identity
Gipr
Glp1r
C.
Oligodendrocytes
Gipr
Glp1r
UMAP2
UMAP1

Supp Fig 5

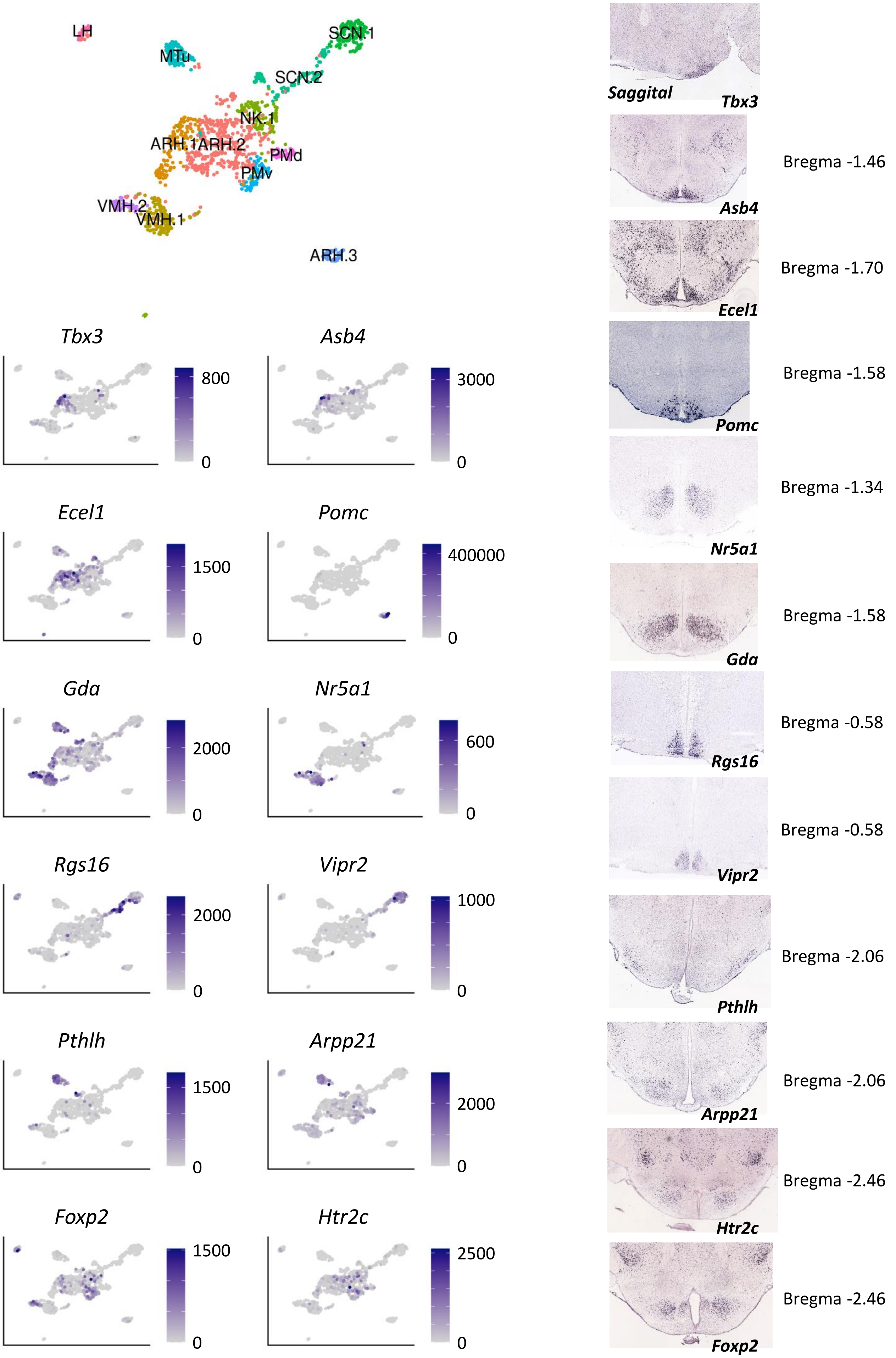
LH
MTu
SCN.1
SCN.2
NK.1
ARH.1
ARH.2
PMd
PMv
VMH.2
VMH.1
ARH.3
Tbx3
800
0
Asb4
3000
0
Ecel1
1500
0
Pomc
400000
0
Gda
2000
0
Nr5a1
600
0
Rgs16
2000
0
Vipr2
1000
0
Pthlh
1500
0
Arpp21
2000
0
Foxp2
1500
0
Htr2c
2500
0
Saggital
Tbx3
Asb4
Bregma -1.46
Ecel1
Bregma -1.70
Pomc
Bregma -1.58
Nr5a1
Bregma -1.34
Gda
Bregma -1.58
Rgs16
Bregma -0.58
Vipr2
Bregma -0.58
Pthlh
Bregma -2.06
Arpp21
Bregma -2.06
Htr2c
Bregma -2.46
Foxp2
Bregma -2.46

Cluster	gene	avg_log2FC Cluster vs Other Clusters	pct. Cells in Cluster	pct. Cells in Other Clusters	p_val	p_val_adj
ARH.2	Fxyd6	0.7265	0.829	0.841	5.87E-19	9.77E-15
ARH.2	Phlda3	0.6125	0.666	0.419	5.18E-18	8.63E-14
ARH.2	Gad2	1.2293	0.639	0.571	4.50E-16	7.48E-12
ARH.2	Aldoc	0.8610	0.658	0.573	1.24E-14	2.06E-10
ARH.2	Slc6a1	0.5287	0.692	0.54	4.43E-12	7.37E-08
ARH.2	Cygb	0.4897	0.705	0.598	4.83E-12	8.03E-08
ARH.2	Slc32a1	0.8597	0.503	0.427	6.09E-12	1.01E-07
ARH.2	Ctxn2	0.5435	0.666	0.585	6.56E-12	1.09E-07
ARH.2	Nrxn3	0.6018	0.855	0.826	1.59E-11	2.65E-07
ARH.2	Cplx1	0.7122	0.521	0.443	2.27E-10	3.78E-06
ARH.2	Gad1	0.8376	0.603	0.503	4.52E-10	7.52E-06
ARH.2	Sncb	0.5778	0.918	0.873	7.05E-10	1.17E-05
ARH.2	Gng13	0.4891	0.589	0.451	6.36E-07	0.010583791
ARH.2	Pnoc	0.7416	0.316	0.182	4.55E-06	0.07562056
ARH.2	Ecel1	0.6281	0.437	0.301	6.12E-05	1
ARH.1	Gaa	1.1921	1	0.953	2.46E-26	4.08E-22
ARH.1	Gabre	1.3537	0.634	0.242	2.28E-22	3.79E-18
ARH.1	Serpina3n	1.9197	0.455	0.111	2.41E-22	4.01E-18
ARH.1	Gpx3	1.3705	0.966	0.629	5.57E-22	9.28E-18
ARH.1	Asb4	2.0420	0.593	0.185	1.37E-16	2.28E-12
ARH.1	Npy1r	1.2476	0.455	0.195	3.47E-15	5.77E-11
ARH.1	Cited1	1.5871	0.662	0.293	3.98E-15	6.61E-11
ARH.1	Irs4	1.4125	0.828	0.433	4.26E-15	7.09E-11
ARH.1	Tbx3	1.4945	0.345	0.053	9.66E-14	1.61E-09
ARH.1	Npy2r	1.1726	0.49	0.084	1.55E-13	2.58E-09
ARH.1	Cartpt	2.9623	0.49	0.294	3.78E-13	6.29E-09
ARH.1	Vgf	1.5056	0.579	0.467	2.07E-09	3.44E-05
ARH.1	Ecel1	1.2689	0.614	0.306	8.81E-08	0.001465083
ARH.1	Gpr101	1.2704	0.572	0.234	2.57E-07	0.004268104
ARH.1	Gal	1.5758	0.359	0.209	0.001463076	1
VMH.1	Nr5a1	2.1233	0.841	0.055	8.55E-117	1.42E-112
VMH.1	Camk1g	1.9548	0.924	0.321	9.92E-93	1.65E-88
VMH.1	Tagln	2.1876	0.864	0.519	1.79E-56	2.98E-52
VMH.1	Tmem35a	2.2159	0.977	0.532	7.63E-56	1.27E-51
VMH.1	Camkv	1.8980	0.985	0.669	1.15E-50	1.91E-46
VMH.1	Galnt16	1.9957	0.97	0.647	4.58E-45	7.63E-41
VMH.1	Nptx1	2.1063	0.917	0.361	5.89E-40	9.81E-36
VMH.1	Ncdn	1.9194	0.985	0.792	3.64E-36	6.06E-32
VMH.1	Lmo3	2.1204	0.924	0.393	5.39E-35	8.97E-31
VMH.1	H2-Q2	2.2146	0.879	0.271	1.26E-33	2.10E-29
VMH.1	Lbhd2	2.3935	0.894	0.318	5.79E-32	9.63E-28
VMH.1	Nptx2	2.0983	0.924	0.194	1.19E-31	1.99E-27
VMH.1	Cnr1	2.1279	0.955	0.414	4.79E-31	7.97E-27
VMH.1	Gda	2.1209	0.977	0.508	1.01E-24	1.68E-20
VMH.1	Fezf1	2.2739	0.811	0.069	5.98E-24	9.94E-20

NK.1	Kcnq1ot1	1.1176	0.984	0.968	1.91E-28	3.18E-24
NK.1	Rab27b	1.2876	0.656	0.701	2.63E-28	4.37E-24
NK.1	Scn9a	1.4952	0.803	0.543	4.51E-26	7.50E-22
NK.1	B3gnt2	1.0502	0.287	0.108	9.52E-24	1.58E-19
NK.1	Hspa5	1.0755	0.885	0.945	1.57E-18	2.61E-14
NK.1	Tmed10	1.0640	0.746	0.776	1.84E-18	3.06E-14
NK.1	Ankrd11	0.9712	0.836	0.769	4.55E-18	7.57E-14
NK.1	Itgb1	0.9983	0.705	0.603	9.15E-17	1.52E-12
NK.1	Dlx5	1.0368	0.32	0.181	1.22E-13	2.03E-09
NK.1	Avp	2.9304	0.205	0.462	3.37E-12	5.61E-08
NK.1	Dlx6os1	1.2360	0.287	0.099	1.52E-11	2.53E-07
NK.1	Plp1	0.9581	0.697	0.401	5.33E-11	8.86E-07
NK.1	Rab3b	1.0310	0.533	0.53	7.13E-11	1.19E-06
NK.1	Rasgrp1	1.0140	0.377	0.371	3.76E-06	0.062587174
NK.1	Gal	1.4129	0.434	0.204	0.008616034	1
SCN.1	Vipr2	1.8925	0.948	0.069	4.07E-138	6.78E-134
SCN.1	Tmem51	1.5690	0.879	0.071	4.32E-127	7.19E-123
SCN.1	Dbi	2.1205	0.974	0.705	5.15E-62	8.56E-58
SCN.1	Prokr2	2.2615	0.94	0.076	7.38E-52	1.23E-47
SCN.1	B3gat2	1.8498	0.983	0.297	1.15E-48	1.91E-44
SCN.1	Pkib	2.7777	1	0.411	2.33E-48	3.87E-44
SCN.1	Ckb	1.5636	1	0.959	1.97E-36	3.28E-32
SCN.1	Tle4	1.4642	0.991	0.629	9.06E-26	1.51E-21
SCN.1	Scg2	1.7071	1	0.939	9.14E-25	1.52E-20
SCN.1	Syt10	2.1170	0.983	0.282	2.41E-22	4.01E-18
SCN.1	Six6	1.9035	0.983	0.281	1.33E-19	2.21E-15
SCN.1	Nnat	1.7086	1	0.853	5.60E-19	9.32E-15
SCN.1	Rorb	1.5073	0.991	0.41	1.46E-09	2.42E-05
SCN.1	C1ql3	1.5440	0.94	0.195	1.32E-06	0.021943907
SCN.1	Cck	1.5488	0.543	0.175	0.001507916	1
SCN.2	Sik1	1.2196	0.648	0.217	1.11E-35	1.84E-31
SCN.2	Pde10a	1.9267	0.907	0.601	1.09E-31	1.81E-27
SCN.2	Avpi1	1.4084	0.778	0.416	4.31E-29	7.17E-25
SCN.2	Rgs16	2.4719	0.769	0.287	4.49E-27	7.48E-23
SCN.2	Rasl11b	1.8631	0.491	0.097	8.03E-26	1.34E-21
SCN.2	Prok2	2.5054	0.333	0.037	6.55E-25	1.09E-20
SCN.2	Rorb	2.0774	0.843	0.427	1.23E-16	2.04E-12
SCN.2	Ppp1r17	1.5178	0.565	0.303	4.27E-12	7.11E-08
SCN.2	Lhx1	1.5184	0.75	0.166	5.41E-12	9.01E-08
SCN.2	Rora	1.2118	0.833	0.559	3.66E-11	6.09E-07
SCN.2	Six3	1.3341	0.991	0.52	1.41E-10	2.34E-06
SCN.2	Nms	1.2943	0.444	0.062	2.21E-09	3.67E-05
SCN.2	Arhgap36	1.1990	0.704	0.332	5.46E-07	0.009083917
SCN.2	Dlk1	1.2926	0.991	0.561	8.68E-07	0.014435092
SCN.2	Avp	1.8937	0.593	0.425	3.61E-05	0.600542239
MTu	Sst	3.7091	0.969	0.228	3.52E-11	5.85E-07
MTu	Xist	2.7175	0.908	0.069	7.27E-10	1.21E-05
MTu	Pthlh	2.3665	0.867	0.075	2.28E-40	3.80E-36

MTu	Otp	2.1783	0.949	0.094	4.51E-09	7.51E-05
MTu	Bcl11b	1.9029	0.939	0.217	4.95E-17	8.24E-13
MTu	Vtn	1.8089	0.867	0.223	1.62E-23	2.69E-19
MTu	Ppp1r1b	1.5134	0.694	0.021	1.05E-84	1.75E-80
MTu	Mef2c	1.4935	0.908	0.471	1.00E-14	1.67E-10
MTu	Ramp1	1.3835	0.745	0.109	5.07E-50	8.44E-46
MTu	Arpp21	1.3213	0.959	0.477	4.57E-12	7.60E-08
MTu	Rnf152	1.2609	0.878	0.362	1.25E-16	2.09E-12
MTu	Rgs5	1.2260	0.786	0.182	9.36E-14	1.56E-09
MTu	Cplx2	1.2123	0.949	0.652	9.29E-17	1.55E-12
MTu	Icam5	1.1949	0.816	0.27	1.38E-22	2.30E-18
MTu	Crym	1.1589	0.531	0.059	2.72E-17	4.53E-13
PMv	Tac1	3.1819	0.536	0.224	8.49E-07	0.014127925
PMv	Calb2	2.5511	0.928	0.549	1.05E-13	1.74E-09
PMv	Foxp2	2.4697	0.754	0.177	1.71E-10	2.84E-06
PMv	Nxph1	2.3556	0.826	0.284	1.51E-14	2.52E-10
PMv	Vsnl1	2.2530	0.986	0.795	1.40E-22	2.33E-18
PMv	Pitx2	2.2430	0.667	0.081	4.87E-10	8.10E-06
PMv	Htr2c	2.2300	0.739	0.25	3.22E-11	5.35E-07
PMv	Ret	2.1920	0.638	0.106	4.60E-21	7.65E-17
PMv	Nr4a2	2.1689	0.667	0.089	3.13E-06	0.052063766
PMv	Prepl	2.1662	1	0.672	5.43E-35	9.03E-31
PMv	Nos1	2.1515	0.812	0.29	2.33E-17	3.88E-13
PMv	Nrn1	2.1130	0.928	0.153	6.89E-06	0.114594439
PMv	Necab1	2.0812	0.768	0.198	4.06E-07	0.006755347
PMv	Ebf3	2.0609	0.478	0.052	7.99E-06	0.13294573
PMv	Nexmif	1.9420	0.957	0.682	8.69E-32	1.45E-27
ARH.3	Pomc	8.6731	0.979	0.662	6.35E-86	1.06E-81
ARH.3	Cga	8.5857	0.375	0.176	1.03E-76	1.71E-72
ARH.3	Epcam	4.1587	1	0.021	9.06E-05	1
ARH.3	Mt1	3.9431	0.958	0.476	6.76E-32	1.12E-27
ARH.3	Oacyl	3.6121	0.521	0.015	1.47E-66	2.44E-62
ARH.3	Btg2	3.4457	1	0.369	1.62E-34	2.70E-30
ARH.3	Ier2	3.4275	0.854	0.2	3.49E-40	5.81E-36
ARH.3	Tgfbr3l	3.3969	0.25	0.034	6.78E-27	1.13E-22
ARH.3	Ascl1	3.1900	0.854	0.054	9.95E-48	1.66E-43
ARH.3	Fos	3.1412	0.896	0.423	9.36E-14	1.56E-09
ARH.3	Junb	3.1108	0.917	0.466	3.50E-20	5.82E-16
ARH.3	H2bc4	3.0715	0.979	0.167	4.87E-58	8.10E-54
ARH.3	Chga	3.0322	1	0.882	1.67E-45	2.78E-41
ARH.3	Nnat	2.8285	0.646	0.874	3.08E-23	5.13E-19
ARH.3	Mt2	2.8105	0.854	0.124	4.21E-32	7.00E-28
VMH.2	Tac1	2.3915	0.872	0.217	0.002958189	1
VMH.2	Bcl11b	1.9542	0.957	0.245	4.90E-08	0.000815414
VMH.2	Gda	1.8519	1	0.538	2.68E-07	0.004454407
VMH.2	Ifi27l2a	1.8336	0.723	0.071	0.000124478	1
VMH.2	Rasgrf2	1.5764	1	0.806	2.91E-14	4.85E-10
VMH.2	Adcyap1	1.4676	0.894	0.308	0.003701353	1

VMH.2	Cdh7	1.4126	0.936	0.311	6.14E-13	1.02E-08
VMH.2	Vcan	1.3533	0.681	0.07	1.13E-12	1.88E-08
VMH.2	Foxp2	1.2896	0.915	0.181	0.008318857	1
VMH.2	Ust	1.2378	0.809	0.159	3.11E-11	5.17E-07
VMH.2	Tppp3	1.2144	0.957	0.69	4.74E-09	7.88E-05
VMH.2	Nrgn	1.2035	1	0.562	0.005143549	1
VMH.2	Xist	1.1867	0.489	0.117	9.99E-55	1.66E-50
VMH.2	Cbln4	1.1620	0.894	0.252	0.000508663	1
VMH.2	Cnr1	1.1501	0.936	0.451	0.000595908	1
PMd	Foxb1	3.0305	0.929	0.008	2.28E-105	3.80E-101
PMd	Serpini1	2.5015	0.952	0.607	1.29E-20	2.14E-16
PMd	Rprm	2.8295	0.548	0.354	1.37E-15	2.28E-11
PMd	Rmst	2.0071	1	0.609	5.32E-15	8.85E-11
PMd	Pcp4	2.6976	1	0.791	8.51E-15	1.42E-10
PMd	Tafa1	2.3006	0.857	0.29	3.75E-13	6.24E-09
PMd	Lhx1os	1.9804	0.976	0.164	4.52E-11	7.52E-07
PMd	Hpca	1.8378	0.976	0.412	1.20E-10	2.00E-06
PMd	Snca	1.7241	0.976	0.668	6.34E-09	0.000105404
PMd	Tmem163	1.6952	0.905	0.342	2.56E-08	0.000426053
PMd	Lhx1	2.0361	0.976	0.189	1.54E-07	0.002566435
PMd	Nxph4	2.6167	0.952	0.047	1.36E-06	0.022574139
PMd	Hopx	1.6875	0.476	0.166	1.68E-06	0.027875245
PMd	Synpr	2.0236	0.476	0.249	2.10E-06	0.034972261
PMd	Cck	2.5178	0.929	0.183	0.002072621	1
LH	Ptpn3	3.4145	1	0.124	1.16E-138	1.93E-134
LH	Ptpn4	3.2294	1	0.606	2.14E-70	3.56E-66
LH	Cit	3.1554	1	0.724	8.97E-65	1.49E-60
LH	Gabra4	3.7560	1	0.369	6.31E-56	1.05E-51
LH	Kcnc2	3.2097	1	0.42	8.28E-44	1.38E-39
LH	Rora	3.6837	1	0.57	1.19E-41	1.98E-37
LH	Slc1a2	4.3872	1	0.328	1.22E-38	2.04E-34
LH	Stum	3.6929	1	0.282	4.20E-36	6.99E-32
LH	Hlf	3.2694	0.971	0.542	1.55E-28	2.58E-24
LH	Ramp3	3.2883	0.588	0.074	1.62E-23	2.70E-19
LH	Ntng1	3.5167	1	0.457	3.44E-17	5.72E-13
LH	Shox2	4.2950	1	0.025	4.10E-14	6.82E-10
LH	Zic1	3.4176	1	0.261	2.75E-13	4.57E-09
LH	Tcf7l2	5.0042	1	0.139	1.35E-08	0.00022482
LH	Cck	3.2997	0.971	0.187	0.000260031	1
